# Evaluation of a Community-Based AI-Assisted Visual Impairment Screening Model for Performance, Operational Efficiency, Acceptability, Feasibility, and Costs: Protocol for a 2-Arm Pragmatic Randomized Controlled Trial

**DOI:** 10.2196/74164

**Published:** 2026-03-02

**Authors:** Yibing Chen, Kai Hui Koh, Jin Wei Clarine Ho, Samantha Min Er Yew, Jocelyn Hui Lin Goh, Elaine Lum, Yih Chung Tham

**Affiliations:** 1 Centre for Innovation and Precision Eye Health, Yong Loo Lin School of Medicine, National University of Singapore, Singapore, Singapore; 2 Department of Ophthalmology, Yong Loo Lin School of Medicine, National University of Singapore, Singapore, Singapore; 3 Singapore Eye Research Institute, Singapore National Eye Centre, The Academia, 20 College Road, Discovery Tower Level 6, Singapore, 169856, Singapore, 65 6576 7298; 4 Centre for Population Health Research & Implementation, SingHealth, Singapore, Singapore

**Keywords:** artificial intelligence, visual impairment, pragmatic randomized controlled trial, retinal imaging, prospective studies

## Abstract

**Background:**

Visual impairment (VI) affects more than 600 million people globally and significantly reduces quality of life. In Singapore, 20% of adults aged 60 years and older (~180,000 people) have VI, a figure expected to double by 2030 due to population aging. While about half of VI cases are due to uncorrected refractive errors, the rest are caused by age-related diseases. The current traditional screening model is a 2-visit, labor-intensive approach with low follow-up rates and frequent unnecessary referrals. Although AI for Disease-related Visual Impairment Screening Using Retinal Imaging, the deep learning model in this study, has demonstrated strong diagnostic performance in retrospective datasets (area under the curve=0.942), key aspects of real-world implementation such as operational efficiency, patient acceptability, workflow feasibility, and cost remain insufficiently studied. As a result, real-world evidence directly comparing artificial intelligence (AI)–assisted and traditional screening pathways is limited.

**Objective:**

This study aims to evaluate the referral accuracy, operational efficiency, acceptability, feasibility, and cost of an AI–assisted screening model compared with the current traditional screening model.

**Methods:**

This study aims to recruit 1000 participants aged 50 years and older using a 2-arm pragmatic randomized controlled trial design. Participants with presenting visual acuity worse than 6/12 (L2) will be randomized 1:1 into either the AI-assisted or traditional screening arms. In the AI-assisted arm, the AI model will analyze retinal photos on-site, with positive cases referred to an optometrist for secondary evaluation. The AI model, previously developed with promising diagnostic accuracy and further validated using community-acquired data, has been integrated with a custom user interface for use in this study. Traditional screening will include pinhole visual acuity, intraocular pressure, slit lamp examination, auto refraction, and retinal photography. All L2 participants will complete a patient-acceptance questionnaire and undergo assessments to determine ground truth.

**Results:**

The study was funded in 2022. Participant recruitment commenced in July 2024, with 487 participants enrolled as of September 14, 2024. Recruitment is ongoing, with study completion anticipated by March 2026 and data analysis expected to begin in April 2026.

**Conclusions:**

This study will provide critical evidence on the clinical utility, feasibility, and cost analysis of AI-assisted VI screening. Our findings may contribute real-world evidence to inform scalable, sustainable screening strategies that enhance efficiency, accuracy, and health system outcomes.

## Introduction

### Background and Rationale

Visual impairment (VI) affects approximately 600 million individuals globally and significantly reduces quality of life, independence, and mobility [1]. In Singapore, one-fifth of adults aged 60 years and older (~180,000 people) experience VI [2]. This figure is expected to nearly double by 2030 due to population aging [1]. Among the VI cases, approximately half result from uncorrected refractive errors, primarily myopia, which can be readily corrected with properly dispensed spectacles available at optical shops. The remaining half are caused by age-related ocular diseases, including cataract, diabetic retinopathy (DR), age-related macular degeneration (AMD), and glaucoma, requiring specialized medical or surgical intervention [3]. Alarmingly, around 70% of older adults with VI in Singapore remain undiagnosed, primarily due to low awareness [4]. Findings from the Singapore Epidemiology of Eye Diseases (SEED) study highlighted that 80% of VI cases among the older adults are treatable [2]. Timely detection through effective vision screening and prompt identification of underlying causes are crucial to prevent disease progression and severe visual loss [5]. Thus, primary prevention and early intervention are essential to mitigate the burden of VI.

The traditional vision screening model commonly used in Singapore involves a 2-visit process. During the first visit, visual acuity (VA) and pinhole tests are conducted, and participants with poor pinhole VA (worse than 6/12) are referred for a second visit on a separate day for additional slit lamp and retinal examinations [6]. While this model provides a structured and thorough screening process, it is relatively manpower-intensive and may require additional effort from participants, potentially impacting follow-up rates and service capacity. As demand for vision screening grows with an aging population, exploring more streamlined and scalable approaches may help enhance accessibility, efficiency, and long-term sustainability.

Artificial intelligence (AI) presents transformative possibilities for health care delivery [7]. Recently, we developed AVIRI (AI for Disease-related Visual Impairment Screening Using Retinal Imaging), a novel deep learning-based algorithm leveraging retinal photography to detect disease-related VI. In a proof-of-concept study published in *Lancet Digital Health* (2021), AVIRI achieved high accuracy (area under the curve [AUC] 0.942) in identifying eye disease-related VI compared with ophthalmologist diagnoses [8]. Although many AI models have shown promising performance on retrospective evaluation, real-world implementation required more than diagnostic accuracy. Successful adoption also depends on workflow feasibility, efficiency, patient acceptability, and cost [9-11]. These aspects of real-world implementations have not been studied in previous AI research. To that end, real-world validation of AI-assisted screening remains limited, with even fewer studies comparing between AI-assisted and traditional screening approaches. To address this gap, we propose an AI-assisted screening model that combines a presenting VA test with AVIRI and will directly compare its performance against traditional screening methods to evaluate efficiency and accuracy in detecting VI.

### Hypothesis and Objectives

This study is designed as a hybrid type 1 effectiveness-implementation trial to evaluate and compare the performance, operational efficiency, acceptability, feasibility, and cost differences between the AI-assisted screening model and the traditional screening model in a community screening setting. A pragmatic randomized controlled trial (pRCT) design will be used to better represent the targeted real-world population. This study will focus on the following specific aims:

Aim 1: To evaluate and compare the referral accuracy between the AI-assisted screening model and traditional screening model for disease-related VI.

Hypothesis 1: The AI-assisted screening model will outperform the traditional screening model in detecting and referring VI cases.

Aim 2: To compare the operational efficiency and acceptability between the AI-assisted screening model and the traditional screening model, and to investigate the feasibility of the AI-assisted screening model.

Hypothesis 2: The AI-assisted screening model will demonstrate greater operational efficiency and acceptability compared to the traditional screening model and will be feasible for implementation in community-based settings.

Aim 3: To compare the overall costs of the AI-assisted screening model and the traditional screening model from a health system perspective.

Hypothesis 3: The AI-assisted screening model will be more cost-effective than the current traditional screening model from a health system perspective.

## Methods

### AI Model Development and Validation

In a proof-of-concept study, AVIRI demonstrated high diagnostic performance, achieving an area under the receiver operating characteristic curve (AUC) of 0.942 (95% CI 0.922‐0.956), with an accuracy of 87.7%, sensitivity of 94.6%, and specificity of 81.3%, compared with the ground truth of ophthalmologists’ clinical diagnoses. External validation using population-based datasets from China, India, and Australia yielded AUCs ranging from 0.859 to 0.935, further supporting AVIRI’s robustness across different populations [8]. While these findings highlight the model’s ability to detect disease-related VI, its real-world performance in community-based settings with undilated eye examinations has not yet been explored.

To address this gap, a previous retrospective evaluation was conducted using 421 retinal images collected in a community setting with the Crystalvue retinal camera (Crystalvue NFC-700; Crystalvue Medical Corporation). This evaluation was part of pilot testing and was not previously published. Multiple domain adaptation strategies were applied to enhance model generalizability, and a ViT-Base architecture, trained on the SEED dataset, was selected as the optimal backbone. This adaptation achieved an AUC of 0.853, specificity of 0.688 (when sensitivity fixed at 80%), and *F*_1_ score of 0.347. Additionally, a user interface was developed to facilitate clinical integration, allowing researchers to input clinical data and generate automated diagnostic reports using AVIRI. These findings provide the foundation for further prospective validation, evaluating AVIRI’s real-world performance in an AI-assisted vision screening model.

### Sample Size Calculation

This study will recruit a total of 1000 participants aged 50 years and older using a 2-arm pRCT design with broad inclusion criteria based solely on age. Of these, 500 participants are targeted to have presenting VA better than 6/12 (L1), a number determined based on operational feasibility in community screenings.

Participants with presenting VA worse than 6/12 (L2) will be randomized to either the AI-assisted or traditional screening model. Sample size estimation for this randomized comparison was based on an assumed accuracy of 70% for the current traditional screening model and prevalence of 18% for any VI, 11% for disease-related VI, and 8% for refractive-error related VI (prevalence estimates were based on the SEED study) [2]. Detecting a clinically meaningful difference of 10% between the 2 screening models with 80% statistical power at a 5% 2-sided type I error requires 250 participants per arm. Therefore, a total of 500 randomized participants (L2) will be included in the primary analysis.

### Randomization

VI is defined according to the United States VI definition as having a presenting VA worse than 20/40 (equivalent to 6/12) in the better-seeing eye [3]. Participants with presenting VA of 6/12 or better (L1) will not be randomized. These participants will receive a basic ocular assessment, including fundus photography and anterior eye imaging. The collected images will not be used in the comparative analysis between the AI-assisted and traditional screening arms.

Participants with presenting VA worse than 6/12 (L2) will be randomized into either the AI-assisted screening arm or the traditional screening arm using a 1:1 allocation ratio. Randomization will be conducted at the patient level, ensuring that both eyes of the same participant are assigned to the same study arm. The randomization sequence will be generated by an independent statistician using the “randomizeR” package in R statistical software. This approach ensures an unbiased allocation process while maintaining the integrity of the study design.

### Study Setting

This study will be conducted at Pioneer Polyclinic, a public primary care clinic located in the western region of Singapore. Polyclinics in Singapore are government-funded health care institutions that provide subsidized outpatient medical services, including acute care, chronic disease management, health screenings, and preventive care. They serve as a key component of the country’s primary health care network and are typically staffed by physicians, nurses, and allied health professionals.

Pioneer Polyclinic is part of the National University Polyclinics (NUP) group under the National University Health System. It serves a multiethnic residential population comprising predominantly older adults and middle-aged individuals, with a substantial proportion managing chronic conditions such as hypertension, diabetes, and hyperlipidemia. The clinic is located in a mature public housing estate, where access to subsidized screening and community-based care is particularly important.

### Data Collection

#### Study Workflow

##### Recruitment and Study Procedures

Recruitment will take place at Pioneer Polyclinic (National University Polyclinics) over an 18-month period. Each participant will attend a single study visit, which is expected to last approximately 30 to 90 minutes. Participants will be recruited through 3 main channels: (1) review of past study records to identify eligible individuals, (2) word-of-mouth referrals, and (3) display of an electronic brochure (e-brochure) on screens within participating polyclinics. For the first recruitment channel, only individuals who meet the study’s eligibility criteria will be invited to participate. Eligible participants must have complete clinical data and retinal imaging records. They must also have previously provided consent to be recontacted.

Both the AI and traditional screening arms will use the same fundus cameras (Crystalvue) and will follow identical equipment calibration protocols according to manufacturer recommendations. All image acquisition will be performed by trained clinical research coordinators who have completed standardized certification in retinal imaging. Refresher training and inter-operator assessments will be conducted prior to and throughout the study to minimize variability.

##### Control Arm

**Figure 1.**
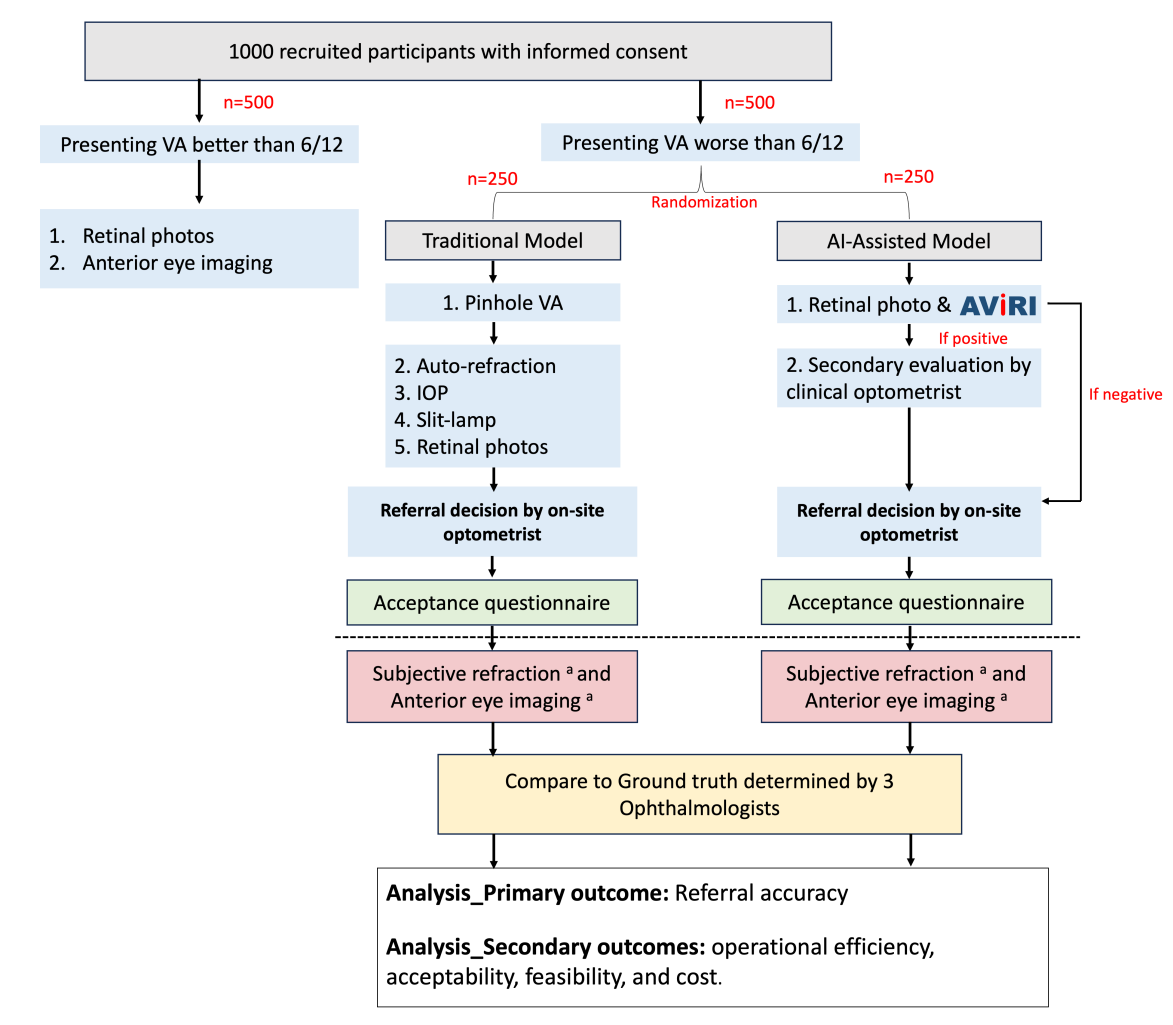
The data collection workflow. ^a^Data to establish the gold standard. AI: artificial intelligence; AVIRI: AI for disease-related visual impairment screening using retinal imaging; IOP: intraocular pressure; VA: visual acuity.

Participants will first sign informed consent before proceeding with any assessment. After being randomized into the traditional model arm, the screening will follow the standard examination workflow used in the traditional screening model, which consists of pinhole VA, intraocular pressure, auto refraction, slit lamp examination, and retinal photography. The fundus photographs will be reviewed by the onsite optometrists, and any images of insufficient quality will be retaken immediately to improve clarity. If image quality remains inadequate, the participant’s ID will be flagged in the study log. Referral decisions will be made by the onsite optometrists, incorporating the findings from all other screening tests. After receiving the referral outcome, participants will be invited to complete a patient-acceptance questionnaire to assess their perceptions and satisfaction with the traditional screening model (Multimedia Appendix 1). Finally, anterior eye photography and subjective refraction will be performed to establish the ground truth status for the referral decision (Figure 1).

##### Intervention Arm

Patients randomized to the AI-assisted arm will undergo retinal photography, with their images analyzed on-site by AVIRI. Fundus images will be checked by onsite optometrists, with poor-quality photographs retaken on the spot. If adequate quality still cannot be obtained, the participant’s ID will be recorded in the study log. The AVIRI report will also include a quality-check notification indicating when an image does not pass its internal quality assessment, alerting the clinical optometrists that the AI-generated results may be affected. If a case is detected by AVIRI as having disease-related VI, the retinal images and AVIRI report will be sent to an assigned clinical optometrist via the institution’s secured Microsoft Teams for further evaluation (Microsoft Corp). Clinical optometrists, who are independent from the onsite screening team, will examine the AI-generated results and make the final referral decision. Only cases identified as positive by the AVIRI model are reviewed by the clinical optometrists, and the referral process follows the same standardized procedure applied across both study arms. Similarly, after the referral decision, participants will complete a patient-acceptance questionnaire to assess their perceptions and satisfaction with the AI-assisted model. Finally, anterior eye photography and subjective refraction will be performed to establish the ground truth status (Figure 1).

### Data Quality Assurance

A senior data coordinator will oversee all data entry procedures to ensure consistency and accuracy throughout the process. Each data entry will be independently verified by a separate data entry personnel to minimize errors and discrepancies. Regular checks will be conducted to ensure that all consented, eligible participants’ data have been properly entered into the system. Additionally, basic tabulation will be performed against the study screening log, and any inconsistencies will be promptly addressed to maintain the integrity of the data. To ensure consistent image quality, all imaging personnel will undergo standardized training and certification in retinal photography. Fundus cameras will be calibrated according to manufacturer guidelines. Images will be captured following a standardized protocol for field of view, focus, and illumination and reviewed in real time. Ungradable images will be repeated onsite and flagged in the data log. Image quality will be graded as good, borderline, or poor. A detailed review process will be implemented to guarantee the accuracy and completeness of the dataset before analysis.

### Data Entry and Storage

Data will be recorded on paper forms and subsequently entered into a password-protected Excel spreadsheet stored on an encrypted storage drive. Slit lamp and retinal photographs will be deidentified and stored in digital format for grading purposes. A digital audio recorder will be used during the patient acceptance questionnaire to facilitate accurate documentation, analysis, and review of discussions. A distinct section in the consent form will be used to obtain participants’ permission for audio recording their responses. The recorded audio data will be transcribed and translated into English (if required) by study team members after the participant’s visit. The data entry process will be supervised by a data coordinator, and all entered data will be independently verified by another data entry personnel.

All data files will be encrypted to ensure data security. Access keys for encryption will be securely stored and made available only to study team members. Data access and key management will adhere to Singhealth’s data retention policy. Case files will be securely stored with restricted access, in accordance with the study delegation log.

Participants’ confidentiality and privacy will be upheld throughout the study, from the initial data collection phase until the secure destruction of personal and sensitive information. Only authorized research team members will have access to research data.

### Outcome Measures

#### Ground Truth Establishment

The ground truth diagnosis will be determined by a panel of 3 board-certified ophthalmologists, who will independently review presenting VA, best-corrected VA data, intraocular pressure measurements, medical history, and anterior and retinal photographs. The reference standard outcome (refer vs not refer) will be determined by majority agreement (2 out of 3 panel experts). Cases without a clear majority (eg, ungradable) will undergo consensus discussion among the experts, and if consensus cannot be reached, a senior ophthalmologist will adjudicate. The adjudicator will not be blinded by the clinical context as it is required to make a referral decision. To ensure the credibility of the pRCT results, the panel of three board-certified ophthalmologists will be blinded to the participants’ group assignments to minimize bias, and they will not participate in any study procedures that could compromise or reveal participants’ randomized group assignments.

#### Primary Outcomes

The primary outcome is the rate of correct referrals based on the reference standard. A correct referral was defined as a case in which the AI or optometrist’s referral decision (refer vs no refer) matched the ground truth outcome determined by the panel of three board-certified ophthalmologists. To evaluate whether the AI-assisted model offers improved referral accuracy compared to the traditional screening model, performance metrics such as accuracy, AUC, sensitivity, and specificity will be calculated and compared for both models. A calibration plot will be used to assess the agreement between the model’s predicted probability of VI and the actual VI outcomes. Performance metrics will also be stratified and analyzed according to the severity of VI. Moderate VI is defined as presenting VA between 6/12 and 6/48, while severe VI is defined as presenting VA of 6/60 or worse [12].

#### Secondary Outcomes

The secondary outcomes of this pRCT include operational efficiency, acceptability, perceived feasibility, and cost evaluation. These secondary outcomes are important for successful implementation and future scalability.

##### Operational Efficiency

This evaluation will assess the effectiveness of the screening workflow by measuring the average time required per patient to complete the entire screening process, providing insights into overall efficiency. The screening capacity will be evaluated by calculating the average number of patients screened per hour. In addition to the average number of patients screened per hour and average time per patient, we will break down the total screening time into specific workflow steps (eg, retinal photo taking, results analysis, review, and referral decision making) to allow a more detailed comparison of operational efficiency between the traditional and AI-assisted arms. These metrics will help determine whether the AI-assisted model improves workflow efficiency compared to the current screening approach.

##### Acceptability

In this study, acceptability is defined as patients’ perception of the AI-assisted screening model in terms of its usability, comfort, and overall satisfaction [13]. All participants randomized to either the AI-assisted or traditional screening arm will be invited to complete a structured questionnaire designed to assess their perception and satisfaction with the screening process. The questionnaire consists of 3 items rated on a 5-point Likert scale, evaluating the overall satisfaction with the screening process [14]. Five dichotomous questions are also included to evaluate factors such as awareness of AI and confidence in results. The questionnaire was developed following established psychometric principles to ensure validity and reliability in assessing patient perspectives [15].

To enhance the accuracy of data collection, trained research coordinators will administer the questionnaire with audio recording after obtaining patients’ consent. The responses will be transcribed verbatim. This approach ensures consistency in responses, minimizes misinterpretation of questions, and allows coordinators to clarify any concerns raised by participants. Missing questionnaire items will be reported as nonresponses. Given the short questionnaire and supervised administration, minimal missing data are expected. However, sensitivity analyses will be conducted if missingness exceeds 5%. Insights gained from this assessment will help refine the implementation of AI-assisted screening models and address potential barriers to patient acceptance.

##### Feasibility

Feasibility is defined as the degree to which the AI-assisted screening model can be effectively implemented within the intended setting [13]. Service providers’ perceptions of feasibility will be assessed through focus group discussions. The results from evaluations of operational efficiency and patient acceptability, as described earlier, will inform the development of discussion questions based on the constructs of the Consolidated Framework for Implementation Research (CFIR), a well-established framework for assessing the implementation of health care innovations. The CFIR provides a structured approach for understanding the factors that influence successful adoption and integration of new interventions within health care settings [16].

Focus group discussions will be conducted with optometrists, each lasting 60 to 90 minutes, with the objective of achieving both data saturation and meaning saturation [17]. All seven clinical optometrists who reviewed the AVIRI output will be invited to join the focus group discussions. The FGD will enable us to capture diverse perspectives and stakeholder experiences with the AI workflow. The focus group discussions will be audio-recorded, transcribed verbatim, and analyzed using framework analysis [18]. Two trained researchers will conduct both inductive and deductive coding, with a third researcher moderating if discrepancies arise after a consensus discussion. The analysis will be interpreted through the lens of the CFIR framework [16]. This in-depth approach will help identify barriers and enablers to the implementation of the AI-assisted screening model from the service providers’ perspectives, facilitate the development of meaningful adjustments to the model, and uncover potential strategies for improving future implementations.

##### Cost Evaluation

We will assess the incremental cost savings of implementing the AI-assisted screening model compared to the traditional screening model using an Activity-Based Costing (ABC) approach. The analysis will include all nonsunk costs incurred up to the referral decision. These costs include labor, materials, supplies, and amortized expenses for technology and space use. Costs will be grouped according to the key activities of each screening model, such as conducting the screening examinations, running the AVIRI algorithm, and generating on-site test results. Fixed costs will be amortized based on the expected useful life of the involved fixed assets. For clinical assessment costs, we will use nonsubsidized billing rates, as these are expected to closely reflect actual costs [19]. As the study involves only a one-time screening visit without longitudinal follow-up, all cost calculations will reflect a cross-sectional time frame, limited to the immediate costs incurred during the single-visit screening encounter.

### Data Analysis Plan

#### Model Performance

The AI model was based on the ViT-base backbone, which was initialized using pretrained weights obtained through supervised learning on the ImageNet-21k dataset [20]. Fine-tuning was carried out for 40 epochs to allow for sufficient learning while reducing the risk of overfitting. The process was performed using a batch size of 16, with a fixed learning rate of 1 × 10⁻⁵, weight decay set at 5 × 10⁻⁴, and the default drop path rate applied. To evaluate the effectiveness of the AI-assisted screening model compared to the traditional screening model in detecting VI, both models will be assessed against the ground truth. Overall accuracy, sensitivity, and specificity will be calculated and compared using *z* tests for 2 proportions. Additionally, AUC and the area under the precision-recall curve will be determined for each model. Differences in AUC and area under the precision-recall curve between the two models will be analyzed using bootstrap resampling. All quantitative analyses for model performance will be conducted using R, including diagnostic accuracy metrics and comparative statistical tests.

#### Acceptability and Operational Efficiency

The acceptance scores, measured as ordinal data, will be compared between the 2 screening models using the Mann-Whitney *U* test. To assess operational efficiency, the average time taken per participant for the entire screening process (efficiency assessment) and the average number of individuals screened per hour (capacity assessment) will be calculated [21]. The time taken for each station will also be assessed. Differences in procedure time and screening volume between the 2 models will be analyzed using *t* tests for mean comparisons. To provide additional insight, subgroup analyses by age and gender will be conducted to further evaluate participant acceptability. Age-based analyses will categorize participants into two groups: those aged 50 years and older, and those aged 70 years and older. Gender-based analyses will compare outcomes between male and female participants. Quantitative data related to acceptability and operational efficiency will be analyzed using R, and qualitative data will be coded and thematically analyzed using NVivo.

#### Cost Evaluation

A decision tree model will be employed to estimate the net costs and potential cost savings of the AI-assisted screening model compared with the traditional screening model. The model starts with a decision node for the screening method, followed by chance nodes incorporating VI prevalence and the probability of a positive or negative referral decision based on the sensitivity and specificity of each screening approach. Costs will be incorporated at the level of each screening arm to capture the resources required, including labor, equipment, and operational expenses associated with a single screening visit. Downstream costs such as treatment or follow-up are not included, as they are assumed to be equivalent across both arms and therefore do not impact the incremental cost comparison.”

**Figure 2.**
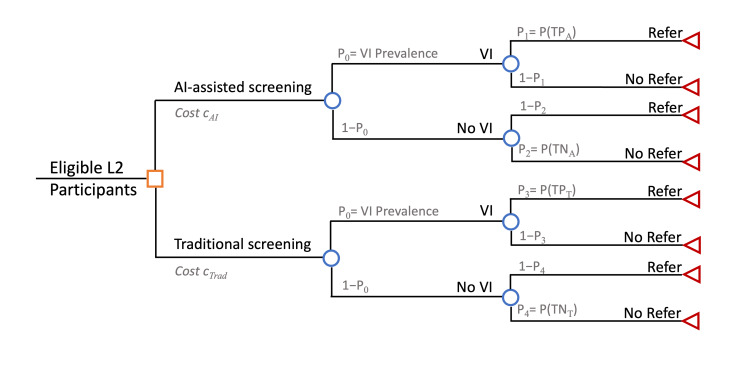
The decision tree structure for cost evaluation. AI: artificial intelligence; Cost *C*_AI_: Total screening cost (per patient) in the AI-assisted arm. Cost *C*_Trad_: Total screening cost (per patient) in the traditional arm. *P*_0_ = VI prevalence: Probability that a participant has visual impairment. *P*_1_ = P(TP_A): Probability of true positive outcome in AI-assisted arm. *P*_2_ = P(TN_A): Probability of true negative outcome in AI-assisted arm. *P*_3_ = P(TP_T): Probability of true positive outcome in traditional arm. *P*_4_ = P(TN_T): Probability of true negative outcome in traditional arm; VI: visual impairment.

Key model parameters will include VI prevalence, screening-related costs, and probability outcomes. The primary outcome will be the patient-level cost incurred under each screening model, restricted to procedures performed up to the referral decision. One-way sensitivity analyses will be conducted on key parameters to assess the robustness of the findings. The underlying hypothesis is that the AI-assisted model is at least as effective as the traditional screening model (Figure 2).

### Ethical Considerations

Written informed consent will be obtained from all participants prior to examination, with a witness present, in accordance with the Declaration of Helsinki. Trained research staff certified in Good Clinical Practice (GCP) will administer the consent process. For participants who do not speak English, the consent document will be provided, and the study procedures will be explained in their preferred language (eg, Malay, Hindi, Tamil, or Mandarin) by study staff. An impartial witness, not involved in the study, will also be present to ensure transparency and comprehension. This study will be conducted in accordance with the Declaration of Helsinki, and ethical approval has been obtained from the SingHealth Centralized Institutional Review Board (CIRB) (2024/2297). Any amendments to the study protocol will be submitted for IRB approval. Data confidentiality will be strictly upheld, and no personal identifiers will be included in any study reports or publications. The study is registered on ClinicalTrials.gov under the registration identifier NCT06877988. L1 participants received an SGD 10 (US $7.90) voucher, while L2 participants received an SGD 30 (US $23.70) voucher as compensation for their participation.

## Results

This study is funded by the National Medical Research Council’s (NMRC) HPHSR Clinician Scientist Award (INV category), awarded in 2022. Participant recruitment and data collection commenced in July 2024. As of September 14, 2024, a total of 487 participants had been recruited. These figures reflect the recruitment status at the time of protocol submission. Recruitment is ongoing, with study completion anticipated by March 2026. Data cleaning and statistical analysis are expected to commence in April 2026, followed by manuscript preparation planned for June 2026.

## Discussion

This study aims to evaluate the effectiveness, feasibility, and cost of an AI-assisted VI screening model compared with the traditional screening model in a real-world community setting. Findings from this work are expected to provide useful insights into the integration of AI tools in primary eye care, particularly regarding operational efficiency, patient acceptability, and economic sustainability. This is especially important given the limited real-world prospective evaluations of AI models in community-based screening. The evidence generated will help address this gap and support future policy and implementation decisions.

This study design offers several strengths that enhance its rigor and potential impact. It adopts a 2-arm pRCT design, increasing the relevance and applicability of findings to routine clinical practice. Most prospective studies involving AI in ophthalmology have focused primarily on evaluating the performance of the AI model itself, with few evaluating the actual workflow and adopting a 2-arm RCT that allows a direct comparison between an AI-assisted screening model and the traditional screening model. For example, Rao et al [22] conducted a prospective study on AI-based detection of referable glaucoma, but the investigation was limited to assessing the AI model’s performance in a research setting. Similarly, Antaki et al [23] prospectively validated an AI model for detecting diabetic retinopathy, yet their study also focused exclusively on AI performance without evaluating its workflow integration or comparing with traditional screening model. In contrast, the head-to-head comparison in our pRCT provides a more comprehensive evaluation of the value-add of AI model in real-world eye screening setting [1] Standardized deployment of the AVIRI model and structured data collection protocols support reproducibility. Additionally, the integration of both quantitative metrics and qualitative feedback, including patient satisfaction and acceptability, provides a comprehensive assessment of model performance and implementation feasibility.

While the pRCT design strengthens internal validity, model-level factors also play a role in shaping screening outcomes in practice. Model performance may also be influenced by the model settings, such as the threshold used to detect the disease-related VI. Adjusting the threshold affects the balance between sensitivity, specificity, and the resulting referral decision. Different community settings may have varying levels of follow-up capacity, workforce availability, and tolerance for false positives or false negatives. Recognizing the impact of these settings is important for interpreting study findings and ensuring that the AI model can be appropriately adapted for real-world implementation.

### Limitations

Despite these strengths, challenges may arise during the implementation process. As a single-site study, generalizability to other health care settings may be limited. Recruitment of participants could pose logistical difficulties, potentially affecting sample size and study power. Variability in image quality and real-world operational constraints could affect model performance. Additionally, assessing feasibility and cost within the study duration may present constraints, requiring assumptions for long-term projections.

To address these challenges, several mitigation strategies will be employed. While generalizability is limited by the single-site design, detailed documentation of the study context will support future multisite applications. Recruitment efforts will be supported through multiple outreach channels and flexible scheduling to enhance participation. If these challenges are appropriately managed, this study will provide critical evidence on the potential role of AI in community-based VI screening and inform future large-scale implementation efforts.

### Conclusion

This study protocol will provide critical insights into the performance, feasibility, and cost of an AI-assisted VI screening model in community settings. By demonstrating its potential advantages over the traditional screening approach, including improved referral accuracy, enhanced operational efficiency, and reduced health care costs, the study outcomes will inform future strategies for implementation. Ultimately, the findings are expected to inform future implementation strategies and contribute to the evidence base for scalable and sustainable approaches to VI screening.

## Supplementary material

10.2196/74164 Multimedia Appendix 1 Subject’s satisfaction and acceptance evaluation questionnaire.

10.2196/74164 Checklist 1 CONSORT-AI checklist [24].

## Abbreviations

AI: artificial intelligence
AUC: area under the curve
AVIRI: AI for Disease-related Visual Impairment Screening Using Retinal Imaging
CFIR: Consolidated Framework for Implementation Research
CONSORT: Consolidated Standards of Reporting Trials
pRCT: pragmatic randomized controlled trial
SEED: Singapore Epidemiology of Eye Diseases
VA: visual acuity
VI: visual impairment
