# Supplementary material for: Evaluation of a Community-Based AI-Assisted Visual Impairment Screening Model for Performance, Operational Efficiency, Acceptability, Feasibility, and Costs: Protocol for a 2-Arm Pragmatic Randomized Controlled Trial
Source: JMIR Res Protoc. 2026 Mar 2;15:e74164. doi: 10.2196/74164 (PMC12954716; doi:10.2196/74164)
Supplement: Multimedia Appendix 1 [file resprot-v15-e74164-s001.docx]

**Subject’s Satisfaction and Acceptance Evaluation Questionnaire**

**STUDY TITLE:** AI-Assisted Visual Impairment Screening Model: Community-based Implementation and Evaluation of Performance, Feasibility and Costs.

**SCREENING NO.:**

**DATE** (DD/MM/YY)**:**

**INVESTIGATOR CODE:**

1. Overall, how satisfied are you with this screening model?
    Very satisfied

Satisfied

Neutral

Dissatisfied

Very dissatisfied

Remarks:

1. How satisfied are you with the waiting time of this screening model?

Very satisfied

Satisfied

Neutral

Dissatisfied

Very dissatisfied

Remarks:

1. How satisfied are you with the flow of this screening model?

Very satisfied

Satisfied

Neutral

Dissatisfied

Very dissatisfied

Remarks:

1. Do you think the results provided by this screening model is reliable?

Yes

No

Don’t know /unsure

Remarks:

1. Do you know what is artificial intelligence (AI)?

Yes (continue with the rest of the questions)

No (stop at this question)

Remarks:

1. Have you heard of the use of AI in eye examination? (when applicable)

Yes

No

Remarks:

1. Do you think AI-assisted screening can reduce overall chair time? (when applicable)

Yes

No

Don’t know /unsure

Remarks:

1. Do you think AI-assisted screening can reduce human effort? (when applicable)

Yes

No

Don’t know /unsure

Remarks:
